# Ureteral Reimplantation with Psoas Bladder Hitch in Adults: A Contemporary Series with Long-Term Followup

**DOI:** 10.1100/2012/379316

**Published:** 2012-07-31

**Authors:** Francesca Manassero, Andrea Mogorovich, Girolamo Fiorini, Giuseppe Di Paola, Maurizio De Maria, Cesare Selli

**Affiliations:** Department of Urology, University of Pisa, Via Paradisa 2, 56124 Pisa, Italy

## Abstract

We retrospectively evaluated our experience with ureteral reimplantation and psoas bladder hitch to restore urinary tract continuity in patients with lower ureteral defects, since long-term data on the outcomes of this procedure have been relatively scarce in the last two decades. The procedure was performed in 24 patients (7 male, 17 female) with a mean age of 54.6 years. The mean ureteral defect length was 4.8 cm (range 3–10), the ureterovesical anastomosis was performed with simplified split-cuff technique in 18 patients, submucosal tunnel in 2, and direct anastomosis without antireflux technique in 2. Mean followup was 53 months (range 12–125), and there were no reinterventions. Postoperative renal imaging was normal in 22 cases (91.6%) and revealed decreased kidney size in 2, 3 patients presented intermittent flank pain, and 5 had sporadic episodes of lower tract UTI but no one pyelonephritis. Psoas hitch ureteral reimplantation can be successfully used for bridging defects of the lower ureter up to 10 cm in length in difficult clinical situations. It is relatively simple to perform, compared to other procedures of ureteral reconstruction, and it provides adequate protection of the upper urinary tract.

## 1. Introduction

The technique of ureteral reimplantation with psoas bladder hitch has belonged to the armamentarium of reconstructive urologic surgery for over a century and is considered an excellent method to restore the urinary tract continuity in patients presenting defects of the lower ureter of different etiologies.

This procedure was originally described in 1896 by Witzel [1], but gained popularity in the 1960s, mostly due to the reports of Zimmerman et al. [2] and Turner-Warwick and Worth [3]. However, following the publication of extensive series from Germany in the 1980s including pediatric and adult cases with a relatively short followup [4, 5], long-term data on the efficacy of this procedure have been relatively scarce in the last two decades [6–8]. 

At the present time, this technique is used by pediatric urologists to stabilize a relatively long submucosal tunnel in the surgical treatment of vesicoureteral reflux and in adult reconstructive urology to bridge lower ureteral defects.

We retrospectively evaluated a contemporary series of adult patients who underwent this reconstructive procedure by the same surgical team with uniform technique, with a mean follow-up approaching 5 years, showing that the ureteral reimplantation with psoas bladder hitch can be favorably used for very long and complicated defects of the lower ureter and should be considered the first reconstructive procedure of the pelvic ureter to attempt.

## 2. Materials and Methods

During the period 2001–2009, ureteral reimplantation with psoas bladder hitch was performed in 24 adult patients (7 male, 17 female) with a mean age of 54.6 years (range 24–74).

The indications were as follows: ureteral injury after gynecological surgical procedures in 8 cases, transitional cell carcinoma of the lower ureter in 5, ureteral strictures due to lithiasis in 5 patients, endometriosis in 3, radiotherapy in one, failed ureteral reimplant with Politano-Leadbetter technique in one, and pelvic recurrence of sigmoid carcinoma in one (Table 1).

In detail the gynecological procedures causing ureteral trauma were abdominal hysterectomy in 6 patients and ovariectomy in 2; ureteral repair was performed between 7 and 20 days from primary surgery.

The procedure was performed with an entirely extraperitoneal approach in 19 cases, while in the remaining 3, the peritoneum was opened either for concomitant surgical procedures or inadequate ureteral mobilization to allow a tension-free anastomosis. Briefly, the bladder was mobilized and both the umbilical arteries were divided and ligated at there origin. The bladder dome was freed from peritoneal attachments with the electrocautery on coagulation mode, and the urachus was divided. An anterior midline cystostomy was performed, taking care not to extend it to the dome, and inserting the left index finger inside the bladder dome, it was possible to mobilize it on the side of ureteral loss in order to reach the psoas minor tendon or muscle, where it was fixed with 2 or 3 sutures in 2-0 polyglycolic acid, taking care not to include the genitofemoral nerve. The ureterovesical anastomosis was then performed, using preferably an antireflux technique.

**Figure 1:**
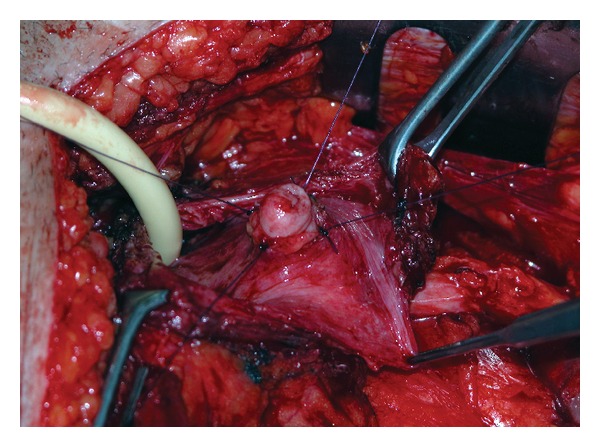
Simplified split-cuff technique of ureteral reimplantation, consisting in the confection of a nipple of the intravesical ureter for a length about the double of its diameter: intraoperative appearance.

**Figure 2:**
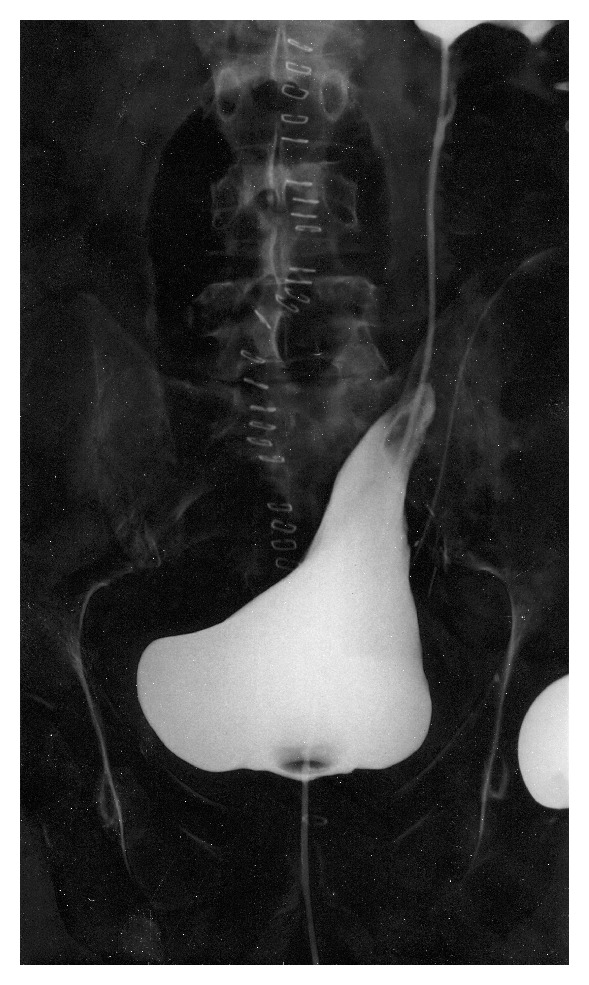
Postoperative cystography following resection of the left pelvic ureter, removed “en bloc” with recurrence of sigmoid carcinoma in a 63-year old man. Extensive bladder mobilization on the left side is evident.

In detail, a simplified split-cuff technique, consisting in nipple-like eversion of the terminal ureter for a length adequate to its caliber was performed in 18 patients (Figure 1), and a submucosal tunnel with Leadbetter-Politano technique was used in 2 cases, while direct anastomosis was performed in 2 cases. Monocryl (poliglecaprone 25) 3-0 sutures were always used for ureteral repair, and double-J stents of adequate length were placed at the end of the procedure and left indwelling for 4 weeks. The bladder catheter was removed after 7 days, following retrograde cystography to rule out extravasation (Figure 2).

**Figure 3:**
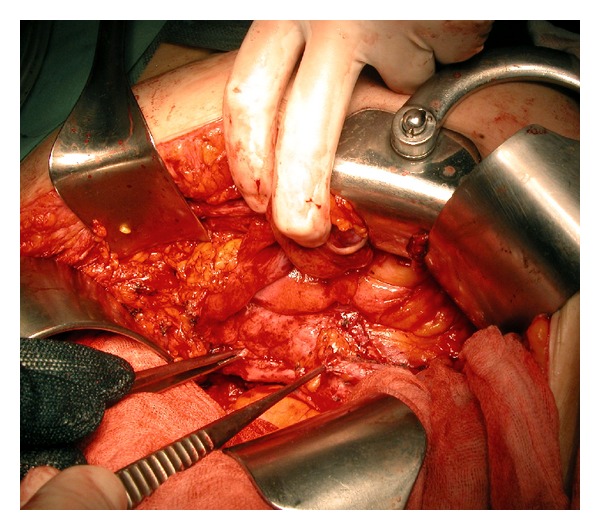
Intraoperative measurement of the ureteral defect length outlined by two forceps.

The mean duration of the entire procedure was 170 minutes (range 130–210), and the mean ureteral defect measured intraoperatively (Figure 3) was 4.8 cm (range 3–10).

## 3. Results

All patients were evaluated with an intravenous pyelography or Uro-CT scan 3 months postoperatively. Subsequently ultrasound imaging of the kidneys and bladder, measurements of serum creatinine and urine culture were performed at yearly intervals. CT scan was performed when necessary for oncological reasons. The mean followup was 53 months (range 12–125). No relevant complications have been documented in the postoperative period (Table 2), and no reinterventions have been necessary. A telephone interview was obtained from all patients during the data collection for the present paper.

Postoperative renal imaging was considered within normal limits in 22 patients (91.6%) and revealed reduced kidney size in 2. Voiding cystography was not performed.

Three patients (12.5%) experienced intermittent flank pain, and 5 had sporadic episodes of lower urinary tract infection (20.8%), but no episodes of pyelonephritis were documented. Serum creatinine was within normal limits in 23 patients, while one woman with a solitary kidney developed mild chronic renal failure (serum creatinine 1.8 mg/dL), but in our opinion this is not ascribable to the procedure.

## 4. Discussion

Tension-free preservation of the urothelial continuity should be always attempted in reconstructive urologic surgery, since it has proved to be superior to ureteral substitution [9], and bladder mobilization represents the more logical solution for bridging gaps of the lower ureter. 

Although the evaluation criteria have changed over time, ureteral reimplantation with bladder psoas hitch has maintained an elevated success rate over time in recent series of adult patients [7, 8].

Some technical details are still controversial, such as the use of nonabsorbable sutures to anchor permanently the bladder to the psoas tendon or muscle. Moreover, refluxing vesicoureteral anastomoses are considered safe in adults by numerous authors [7], but this view is not uniformly shared.

We have found particularly satisfactory to reimplant the ureters with a simplified split-cuff technique, consisting in the confection of a nipple of the terminal ureter for a length about double of its diameter, which does not cause excessive shortening, is technically simple and does cause long-term obstruction. The latter has been recently documented in orthotopic neobladders with the traditional split-cuff technique where a longer segment of the ureter is incised and everted [10].

When ureteral reimplantation is performed after distal ureterectomy for transitional cell tumors, the simplified split cuff facilitates follow-up ureteroscopy, when needed.

In our experience, the functional results of ureteral reimplantation with bladder psoas hitch have been rewarding with long-term followup. However, we observed in 3 cases intermittent lumbar pain on the operated side, first reported by Ahn and Loughlin [7], whose etiology is not entirely clear. We failed to document cases of painful “psoas syndrome” which occurs when the genitofemoral nerve is trapped, particularly when using nonabsorbable sutures. In the present series, no cases of bladder dysfunction were recorded after psoas hitch, and we have reason to believe that complete mobilization of the bladder dome allows an even distribution of tension and effective contraction during voiding.

The feasibility of performing ureteral reimplantation associated with psoas hitch using a robotic-assisted laparoscopic approach has been also reported [11], and it is likely that the use of this technique will expand in the next few years, since it partially overcomes the technical difficulties of creating laparoscopically a nonrefluxing ureteral reimplant.

In conclusion, ureteral reimplantation with psoas bladder hitch can be successfully used for bridging defects of the lower third of the ureter up to 10 cm in length even in difficult clinical situations. It is relatively easy to perform and, coupled with simplified split-cuff antireflux technique, offers adequate protection of the upper urinary tract, and it should be always attempted first in reconstructive procedures of the pelvic ureter.

## Figures and Tables

**Table 1:** Patients' characteristics.

Mean age at surgery (yr)	54.6 (range 24–74)
Gender	
Female (*n*)	17
Male (*n*)	7
Causes of ureteral defect (*n*)	
Gynecological procedures	8
Transitional cell carcinoma of lower ureter	5
Lithiasis	5
Endometriosis	3
Radiotherapy	1
Failed ureteral reimplant	1
Pelvic recurrence of sigmoid cancer	1
Type of ureteral anastomosis (*n*)	
Simplified split cuff	18
Submucosal tunnel	2
Direct	2

**Table 2:** Complications and outcomes.

Reinterventions	0
Reduced kidney size (%)	2 (8.3%)
Intermittent flank pain	3 (12.5%)
Lower UTI	5 (20.8%)
Mild renal failure in solitary kidney	1 (4.1%)
